# Emerging role for interferons in respiratory viral infections and childhood asthma

**DOI:** 10.3389/fimmu.2023.1109001

**Published:** 2023-02-21

**Authors:** Anthony Bosco

**Affiliations:** ^1^ Asthma and Airway Disease Research Center, University of Arizona, Tucson, AZ, United States; ^2^ Department of Immunobiology, The University of Arizona College of Medicine, Tucson, AZ, United States

**Keywords:** rhinovirus, respiratory syncytial virus, childhood asthma, interferons, innate immunity, trained immunity, systems biology

## Abstract

Respiratory syncytial virus (RSV) and Rhinovirus (RV) infections are major triggers of severe lower respiratory illnesses (sLRI) in infants and children and are strongly associated with the subsequent development of asthma. Decades of research has focused on the role of type I interferons in antiviral immunity and ensuing airway diseases, however, recent findings have highlighted several novel aspects of the interferon response that merit further investigation. In this perspective, we discuss emerging roles of type I interferons in the pathogenesis of sLRI in children. We propose that variations in interferon response patterns exist as discrete endotypes, which operate locally in the airways and systemically through a lung-blood-bone marrow axis. We discuss new insights into the role of interferons in immune training, bacterial lysate immunotherapy, and allergen-specific immunotherapy. Interferons play complex and diverse roles in the pathogenesis of sLRI and later asthma, providing new directions for mechanistic studies and drug development.

## Introduction

Bronchiolitis and early wheezing are amongst the first signs that a child is on a trajectory towards asthma (1). RSV is the main causative agent of bronchiolitis and wheezing in the first year of life, and RV becomes the dominant causative agent of both illnesses after age 1 (2). Whilst sLRI with either RSV or RV are strongly associated with later asthma and recurrent wheeze, the odds ratios are much stronger for RV (1, 3–9). Moreover, it is now recognized that bronchiolitis is a heterogeneous condition comprising distinct phenotypes (10). Risk for recurrent wheeze and asthma following severe bronchiolitis in infancy was greatest in the subgroup with RV infection, concurrent wheeze, and a history of wheezing and/or eczema (11, 12).

RV-induced wheezing in the first three years of life is the strongest known objective risk factor for school-age asthma (13). Once asthma is established, RV triggers most exacerbations in both children and adults (14). However, RV infections are ubiquitous and routinely detected in the absence of asthma symptoms (6, 15). This suggests that additional disease co-factors act in combination with RV to trigger asthma. Notably, RV are genetically diverse viruses that are classified into three species and more than 170 strains, and RV-C and RV-A species are more pathogenic than RV-B (14). Risk for development of childhood asthma following RV wheezing is markedly elevated by asthma risk alleles on chromosome 17q21 (16). Early aeroallergen sensitization acts in concert with RV wheezing to substantially increase asthma risk (13, 17, 18), and risk for severe asthma exacerbations among sensitized children is greatest in combination with household allergen exposure (19). Finally, detection of pathogenic bacteria in the airway microbiome is associated with infection spread from the upper to lower airways and risk for subsequent asthma (20).

## Role of deficient interferons in RV infections and asthma

Seminal studies by Wark and coworkers demonstrated that type I and III interferon responses were deficient in bronchial epithelial cells obtained from adults with atopic asthma following RV infection *ex vivo*, leading to increased viral replication and exaggerated secondary responses (21, 22). Moreover, prospective cohort studies have found that the capacity to produce type I and III interferon subtypes in cord blood mononuclear cells (CBMC) was associated with the development of febrile sLRI and persistent wheeze (23). This suggested impaired innate immunity underpins susceptibility to RV/asthma. However, these findings are controversial for several reasons. First, deficient interferon responses were found in some subjects with asthma but not others (24, 25). Second, a clinical trial that administered inhaled interferon-beta therapy at the first signs of a cold failed to prevent worsening of asthma symptoms (26). Third, *in vivo* studies in children with viral bronchiolitis or RV-induced asthma exacerbations found that type I/III interferon responses were increased in the airways and correlated with symptom expression (27–29). Notably, interferon responses can initially be deficient or delayed in subjects with asthma, leading to higher viral loads, which subsequently drive upregulation of interferon responses (30, 31), suggesting that variations in the findings between studies might reflect in part variations in the time course of infection or the site and timing of sample collection (32).

## Interferon response patterns as discrete endotypes

The role of interferons in respiratory infections has primarily been assessed *via* group-wise comparisons between subjects stratified on clinical features. This approach is limited, because it is now recognized that viral bronchiolitis, wheezing, and asthma are highly complex and heterogeneous conditions that are characterized by discrete subgroups with distinct underlying pathologic mechanisms. The advent of molecular profiling technologies now enables the unbiased discovery of endotypes from high dimensional data. We employed this approach to explore immune mechanisms underlying the pathogenesis of severe RSV- or RV-induced bronchiolitis in hospitalized infants and children (33). Through cluster analysis of RNA-Seq profiles in PBMC, we showed the responses could be divided into two molecular phenotypes, which were largely characterized by the presence or absence of IFN-related pathways. Notably, our findings are similar to Turi and coworkers who identified two major patterns of nasal immune responses in infants during their first RSV- or RV-induced acute respiratory illness (34). Together, these data suggest that the two patterns represent discrete endotypes.

The concept of viral bronchiolitis endotypes was recently explored using an integrated, multi-omics approach (35, 36). The authors employed similarity network fusion for data integration and identified four distinct subgroups of RSV bronchiolitis. The first subgroup-A had solo-RSV infection and intermediate IFN-α and IFN-γ responses. Subgroup-B had RV coinfection, higher IFN-α and -γ responses, and higher proportions of parental asthma, IgE sensitization, *S. pneumoniae* and *M. catarrhalis*. Subgroup-C had a history of antibiotic use, mixed microbiome profile and low interferon responses. Subgroup-D had low parental asthma/IgE levels, high abundance of *M. catarrhalis* and an IL-6 inflammatory profile. Importantly, the endotypes had differential susceptibility for the development of childhood asthma, with the interferon-high subgroup-B displaying the greatest risk. Employing the same approach to RV-induced bronchiolitis, the authors identified four endotypes, which differed based on RV type, T2 inflammation, and microbiome profile. Notably, children with endotype-D (RV-C, *Moraxella*-dominant microbiota, T2 high) had increased risk for the development of recurrent wheeze and asthma. Together, these integrated, multi-omics studies demonstrate that infants with viral bronchiolitis can be divided into discrete endotypes that vary based on viral etiology, clinical and microbiome profiles, the nature and intensity of immune response (type I interferon, T2) and risk for later asthma and wheeze.

We utilized nasal swab transcriptomics to explore inflammatory mechanisms operating in children who presented to the emergency department with severe RV-induced exacerbations of asthma/wheezing (37). We found that the expression profiles were heterogeneous and could be divided into two molecular phenotypes. The first phenotype was characterized by upregulation of T1/type I interferon responses and the second phenotype lacked an interferon signature and instead was characterized by upregulation of cytokine and growth factor signaling pathways (e.g. EGF, IL-4R, IL-6, IL-10, TGF-β) and downregulation of IFN-γ (37). Reconstruction of the gene network employing prior experimental evidence identified IRF7 as the dominant hub connecting interferon-mediated antiviral responses, and accordingly we designated the two subgroups as IRF7hi versus IRF7lo phenotypes (37, 38). Notably, knockdown of IRF7 in airway epithelial cells diminished RV-induced antiviral responses and increased IL-33 responses, suggesting that IRF7 controls the balance between antiviral immunity and T2 inflammation (39).

A limitation of studying children presenting to emergency departments is that it is not possible to control for variations in the time-lag between the onset of infection and collection of biological samples. To address this issue, Altman and coworkers followed exacerbation-prone asthmatic children and used daily symptom monitoring to capture children at the onset of cold symptoms (40). Gene expression was profiled in nasal lavage cells at three-time intervals (baseline, 0-3 days, 4 – 6 days post symptom onset) to identify patterns underlying progression to exacerbations. Employing systems scale analyses, they showed that amongst virus positive children, those who experienced exacerbations were characterized by upregulation of a series of modules enriched with SMAD3 signaling, eosinophil activation, extracellular matrix proteins, epidermal growth factor signaling, type I interferon response, Cilia/IL-33 response, and T2 inflammation. Importantly, type I interferon responses peaked at 2 days after the onset of cold symptoms and was more intense among virus positive children who progressed to exacerbation. They also found children with a high ratio of T2 inflammation to type I interferon gene expression at baseline had a shorter time to exacerbation, suggesting that an imbalance between type I interferons and T2 inflammation underlies disease risk.

We established a unique experimental model to investigate immune responses to the RV mimic attenuated Mengovirus (41, 42). The model entailed a comparison between rat strains manifesting high (BN) verses low (PVG) susceptibility to experimental asthma. In naïve animals, virus infection resulted in upregulation of IRF7/interferon responses in PVG, in contrast to BN, which lacked an IRF7/interferon signature and instead upregulated IL-25 and IL-33 expression (42). This suggested the rat model might mirror to some extent the IRF7 phenotypes we observed in children. Notably, sensitization to allergen followed by virus/allergen coexposure in low-risk PVG resulted in the rapid influx of plasmacytoid dendritic cells (pDC) into the airway draining lymph nodes, and rapid and transient airways inflammation alongside IRF7 gene network formation in the lung (43). In contrast, virus/allergen coexposure in high-risk BN unleashed a severe airways/lung eosinophil/ILC2 inflammatory response that failed to resolve out to nine days post infection. Recruitment of pDC was delayed and diminished in the airway draining lymph nodes in BN, and there was a complete absence of IRF7 gene networks in the lung. The presence versus absence of IRF7 gene network structures in the rat model prompted us to investigate personal gene network structures in our nasal transcriptomics data from children with severe exacerbations (44). We observed two major gene network structures in the data centered around IRF7/type I interferons and FCER1G, and we showed that the ratio of interferon- and FCER1G-associated gene network patterns was predictive of recurrence, with low interferon being associated with increased risk of readmission. Back to the rat model, an interesting hallmark of the BN response to virus/allergen coexposure was upregulation of a lung module enriched with TGF-β/SMAD3 signaling and extracellular matrix/collagen proteins (43). Of note, the expression intensity of SMAD3 gene networks in exacerbation-prone asthmatic children interacts with specific pathogenic bacteria in the airway microbiome to increase or decrease risk for severe exacerbations (40, 45).

## Interferon response patterns and the lung-blood-bone marrow axis

**Figure 1:**
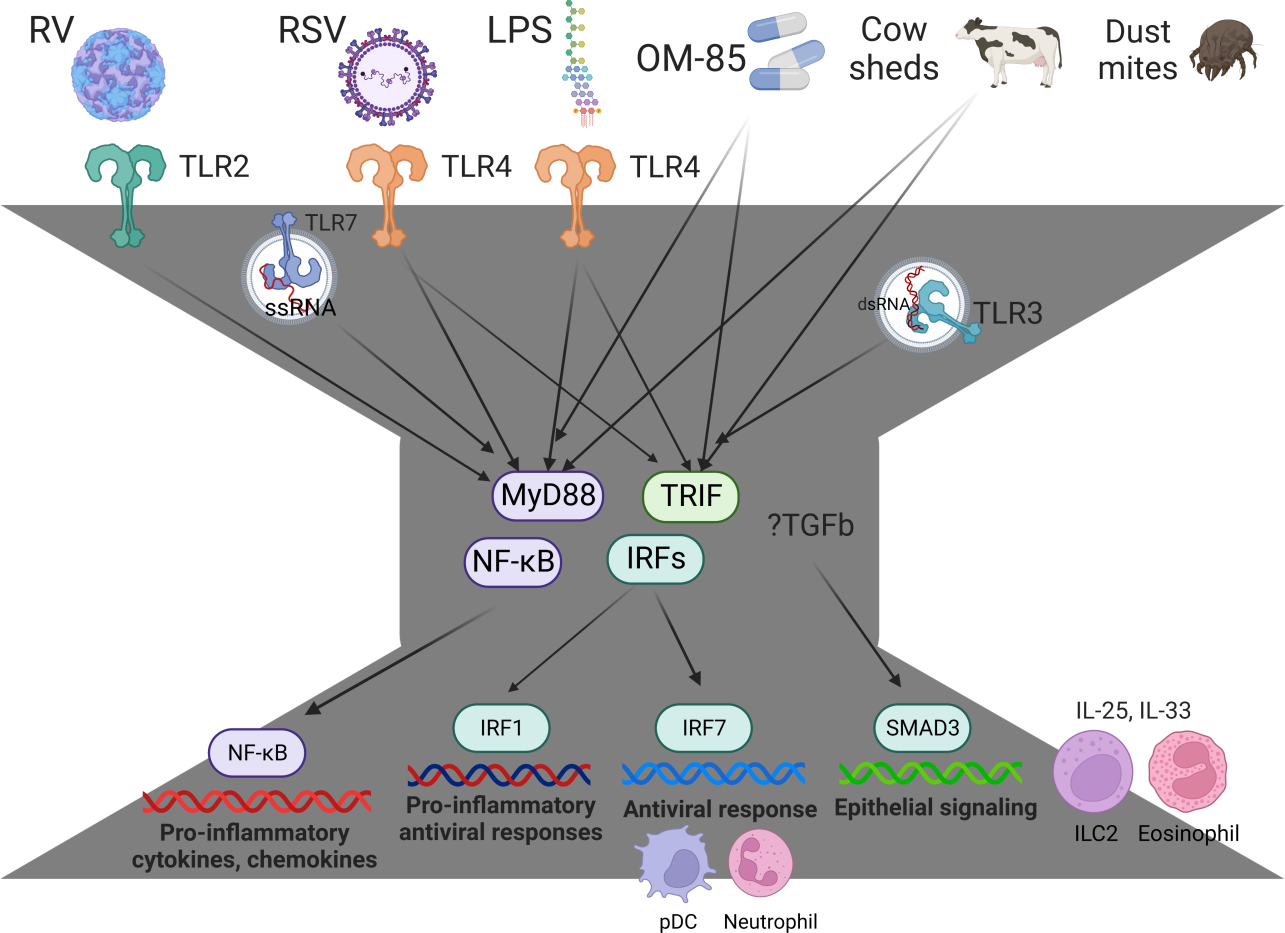
Innate immune responses and immune training are governed by a bow-tie architecture. The bow-tie is a three-layered ordered control system comprising an input layer, a core, and an output layer. The input layer is comprised of pathogen-recognition receptors (e.g. TLR2, TLR3, TLR4, TLR7, MDA5, RIGI), and this layer acts as a sensor for exposure to a broad range of conserved molecular structures derived from viruses, bacteria, allergens, and cow sheds. The core consists of essential, non-redundant components that act as signal integrators or transmitters, such as Myd88, TRIF, and members of the NFκB and IRF family of transcription factors. The output layer consists of a suite of proinflammatory cytokines, chemokines, and antiviral proteins, which mediate immune effector functions. The core components Myd88/TRIF are essential for innate immunity, and these pathways mediate the protective effects of Amish house dust and OM-85 (46, 47). With regards to the output layer, NFκB mediates proinflammatory responses (e.g. IL-1b, IL-6, IL-8, Tnf), IRF7 mediates antiviral responses (e.g. Mx1, ISG20, OASL, RSAD2 (39)) and IRF1 mediates proinflammatory antiviral responses (CXCL9, CXCL10, CXCL11 (48)). RV-induced exacerbations are associated with upregulation of SMAD3 signaling and exaggerated IL-25 and IL-33 responses (40, 45, 49, 50). IRF7hi responders rapidly mobilize pDC into the response and operate through a lung-blood-bone marrow axis (43). IRF7lo responders have increased TGF-β/SMAD3 signaling, exaggerated T2 inflammation that fails to resolve (IL-25, IL-33, eosinophils, ILC2), and prolonged symptoms (37, 42, 43). Figure created with BioRender.com.

Innate immune responses to respiratory viruses begin in the airway epithelium and are governed by a bow-tie architecture (**Figure 1**) (51, 52). Briefly, pathogen-recognition receptors bind to viral proteins and nucleic acids, triggering intracellular signaling cascades that converge on the NF-κB and IRF family of transcription factors to activate their respective proinflammatory and antiviral gene programs (14, 52). The inflammatory milieu in the airways signals to the bone marrow, in order to preprogram myeloid populations and their progenitors, arming them for enhanced effector function as they enter the circulation *en route* to the airways (53, 54). We investigated the lung-blood-bone marrow axis hypothesis in our experimental rat model. Strikingly, we found that IRF7 gene networks were mobilized in the lung and bone marrow of PVG but were completely absent from both compartments in BN (43). We also found an imbalance between type I interferon signaling and T2 inflammation in the bone marrow of PVG versus BN in sensitized animals at baseline, prior to challenge. Malinczak and coworkers found that bone marrow-derived dendritic cells (BMDC) obtained from mice after neonatal RSV infection had enhanced TSLP responses, resulting in exaggerated IL-13 and IL-17A T cell responses to allergen exposure (55). Moreover, BMDC from TSLP deficient mice had enhanced interferon-beta responses to RSV and the TLR agonists Poly-IC and LPS. Together, these data suggest that the balance between T2 inflammation and type I interferon/IRF7 responses may stem from the bone marrow, far from the site of peripheral inflammation, as reported in other contexts (56). They also highlight the potential for new biologic drugs targeting TSLP (57, 58) and the downstream effectors IL-13 and IL-17A (59) to attenuate inflammatory responses to RSV infection in early life.

## Lessons from immune training and bacterial immunotherapy

Trained immunity is the concept that exposure to microbial stimuli (e.g. vaccines, infections, microbiome) results in the long- term functional reprogramming of innate immune cells, which provides enhanced, non-specific protection from subsequent encounters with the same or an unrelated pathogen/stimulus. For example, neonatal BCG vaccination provides protection from respiratory infections (60). An elegant example of immune training is the strong protection from asthma and allergies provided by exposure to traditional farming environments (46, 61). Trained immunity was initially thought to be mediated by mature myeloid cells, which was confusing because myeloid cells have an average half-life of 5–7 days and the effects of trained immunity can persist for months or years (62). However, as noted above, it is now recognized that hematopoietic progenitor cells in the bone marrow act as sensors for peripheral inflammation, which enhances myelopoiesis and preprograms myeloid lineage cells for protective trained immunity (54, 63). Importantly, trained immunity can be induced by therapeutics derived from microbial products. For example, OM-85 and MV130 are formations derived from a cocktail of bacteria (e.g. *Haemophilus influenzae*, *Streptococcus pneumoniae*, *Staphylococcus aureus*, *Klebsiella pneumoniae*, *Moraxella catarrhalis)* that can protect children from viral wheezing (64–66). Accumulating data suggests that type I interferons play a central role in immune training. For example, BCG vaccination upregulates type I and II interferon responses in PBMC, induces long-term DNA methylation changes in the IRF7 promoter in circulating monocytes, and upregulates IRF7 expression in bone marrow stem cells (67, 68). Moreover, IRF7 gene networks are upregulated in the blood of children exposed to Amish house dust, which modulates innate immunity through MyD88- and TRIF-dependent pathways (46). OM-85 treatment dampens proinflammatory responses to a broad range of stimuli, including LPS (69, 70), aeroallergen (47, 71), respiratory viral infections (72, 73), and virus/allergen coexposure (43) and enhances interferon responses in a TRIF-dependent manner (69, 70, 72–74).

## Lessons from allergen immunotherapy

Aeroallergen sensitization in early life synergizes with RV wheezing to drive asthma development (17, 18). Moreover, treatment of asthmatic children with Omalizumab enhances interferon production by pDC (75) and markedly reduces the frequency of exacerbations (76). This provides a rationale for treating high risk children with allergen immunotherapy to reduce the risk of asthma development and/or the frequency of exacerbations (77). Whilst allergen immunotherapy is effective at achieving disease modification and sustained unresponsiveness in allergic rhinitis, it usually requires three years of treatment (78). We recently explored the underlying mechanisms in a cohort of adults with severe allergic rhinitis (79). We found that subcutaneous house dust mite allergen immunotherapy induced the progressive rewiring and integration of mite-driven Th2 gene networks in CD4 T cells with type I interferon networks, suggesting that type I interferons attenuate Th2 responses *via* cross-regulation (79). We observed similar mechanisms operating in peanut allergic children who acquired remission following probiotic plus peanut oral immunotherapy (80). Notably, analysis of dust-mite-driven T-helper memory responses with single cell profiling technologies has unveiled a novel subset of T-helper memory cells that feature a type I interferon response signature (81). Importantly, this novel T-helper memory subset was depleted in allergic subjects, suggesting it may dampen allergic responses.

## Interferon responses to viral versus bacterial stimuli

Wheezy illnesses in young children are associated with both bacterial and viral infections (82). Whilst the protective role of type I interferons in viral infections is well-established, their role in bacterial infections can be beneficial or detrimental, depending on the specific pathogen encountered and the balance between their capacity to exacerbate inflammation or promote tissue homeostasis (83). Current guidelines do not recommend using antibiotics to treat asthma-like episodes in young children, however they are widely prescribed for this purpose. Azithromycin is a macrolide antibiotic that has antibacterial, anti-inflammatory, and antiviral properties. For instance, azithromycin enhances type I and III interferon responses in airway epithelial cells infected with rhinovirus (84). Notably, azithromycin treatment reduces the duration of asthma-like episodes in young children (85) and the frequency of asthma exacerbations in adults (86), but it does not have any long-term beneficial effects on the prevention of recurrent wheeze following severe RSV bronchiolitis (87). Azithromycin also has effects on the gut microbiota (88), and the composition of the airway microbiota modifies the effect of azithromycin on asthma-like illnesses in children (89), and therefore it is likely that the efficacy of azithromycin therapy depends on the specific pathogenic and commensal bacteria and the viral trigger.

We profiled innate immune responses in cord blood mononuclear cells (CBMC) to bacterial LPS and viral nucleic acid sensing pathways (Poly(I:C), Imiquimod) from a birth cohort at high-risk for allergies/asthma (90). Strikingly, we found that LPS- but not Poly(I:C)/Imiquimod-induced interferon responses at birth were predictive of sLRI in the first year of life. Gene networks and master regulators were inferred from the data using a systems biology approach, unveiling IRF1 as a driver of the LPS-induced interferon response. This finding was interesting given that IRF1 drives proinflammatory responses downstream of STAT1 signaling (48) and was correlated with proinflammatory cytokines and chemokines in our data (90). We also compared innate immune responses in matching CBMC/PBMC samples collected at birth versus age 5. We found that LPS-induced interferon responses were upregulated at age 5, however, the role of IRF1 was replaced with other members of the IRF family, including IRF7, suggesting that this pathway is subject to strong developmental rewiring (90). Illy and coworkers investigated the relationship between LPS response patterns at age 1 with asthma risk alleles on 17q21 and asthma/wheeze. They identified three distinct patterns of LPS responses, which were characterized by low, intermediate, or high levels of detectable effector cytokines. They found that risk for wheeze at age 1 and asthma at age 6 in children who carry risk alleles on 17q21 was only observed in the subgroup with low LPS responses (91). Through complex statistical and mediation analyses, they additionally found that oral (farm milk) but not inhaled (barns, stables) exposures impact on the composition of the early gut microbiome, which in turn drives robust LPS responses and mitigates asthma risk in children who carry 17q21 risk alleles.

We evaluated the capacity of OM-85 treatment to reprogram innate immune function in high-risk infants and provide protection from sLRI (70). We found that OM-85 treated children had a longer time to first sLRI, a reduced cumulative frequency of sLRI and reduced number of symptomatic days. Moreover, OM-85 treatment dampened proinflammatory responses and boosted interferon responses to LPS but not poly-IC. Employing differential coexpression analysis, we showed that OM-85 treatment rewired the correlation patterns between TLR4 and interferon signaling pathways, by strengthening the correlation between TLR4 and IFN-β/IRF7 and abolishing the correlation between IRF7 and IRF1. These data suggest that OM-85 rewires the core pathways of the bow-tie architecture that connect input with output signals.

## Discussion

**Figure 2:**
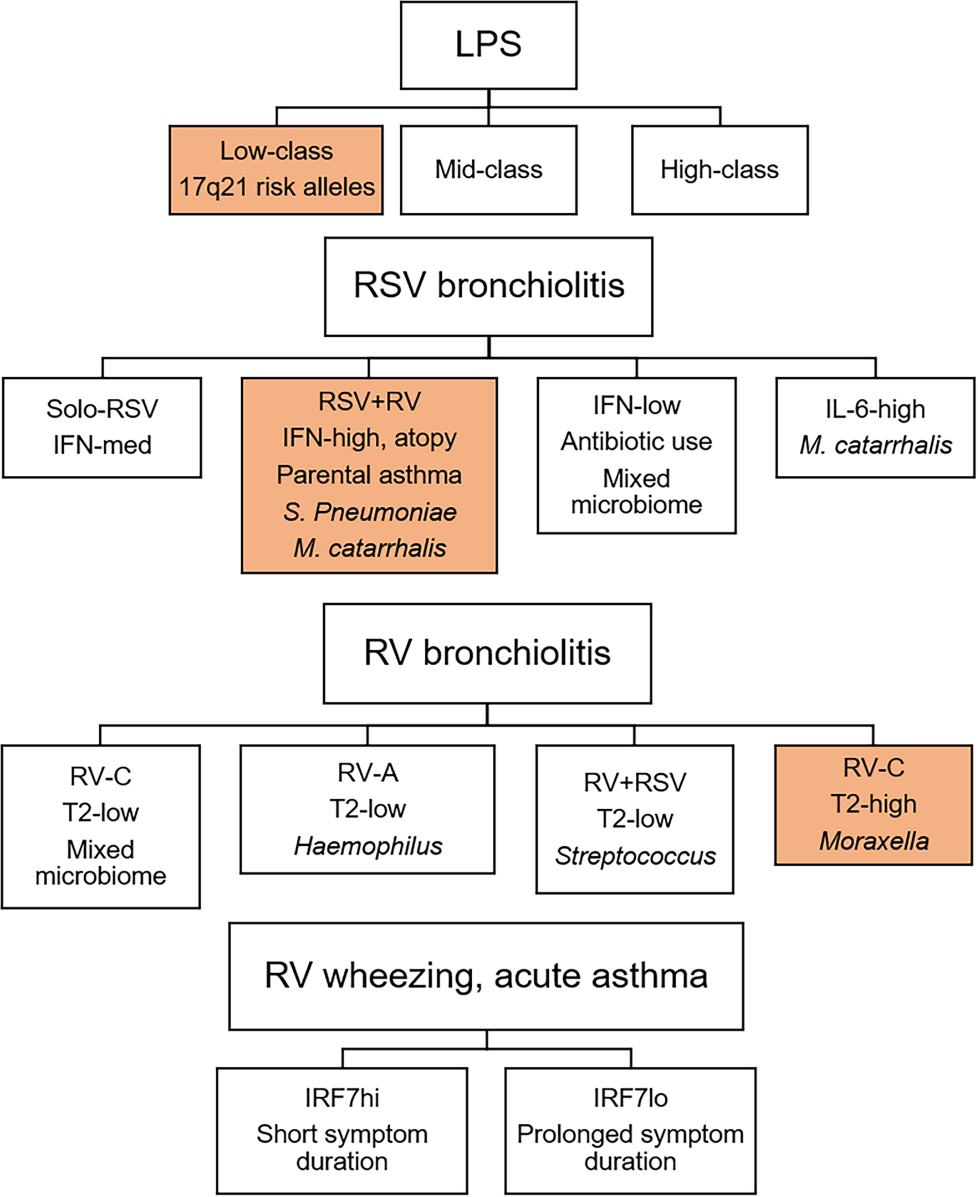
Innate immune response endotypes of viral bronchiolitis, wheezing, and childhood asthma exacerbations. Innate immune response patterns are heterogeneous and can be divided into discrete endotypes. Importantly, response profiles to LPS, RSV bronchiolitis, and RV bronchiolitis are differentially associated with later asthma (35, 36, 91). Children with innate immune response profiles shaded in orange are at increased risk for subsequent asthma. In older children, IRF7 phenotypes during RV-induced wheezing or asthma exacerbations determine symptom duration (37).

Viral bronchiolitis, wheezing, and childhood asthma exacerbations are heterogeneous conditions that can be divided into discrete subgroups (**Figure 2**). We propose variations in interferon response patterns exist as discrete endotypes that operate locally and systematically though a lung-blood-bone marrow axis. Accumulating data suggests that immune training, bacterial lysate immunotherapy, and allergen immunotherapy share a common mechanism of action that is mediated in part by reprogramming type I interferon responses. Emerging data also demonstrates that viruses and pathogenic bacteria interact with host immune response patterns to drive sLRI and asthma development. Furthering our understanding of the complex interactions between viruses, bacteria, and innate immunity will lead to new approaches for the early identification and intervention of high-risk infants.

## Author contributions

AB drafted and edited the manuscript. The author confirms being the sole contributor of this work and has approved it for publication.
